# Exploring Persistent Apical Periodontitis in Humans: Integrative Genetic, Histological and Microbiological Perspectives for Translational Research

**DOI:** 10.1111/iej.70070

**Published:** 2025-12-12

**Authors:** Igor Bassi Ferreira Petean, Alice Corrêa Silva‐Sousa, Rafael Verardino de Camargo, Yara Terezinha Corrêa Silva‐Sousa, Fernanda Gonçalves Basso, André Pitondo‐Silva, Francisco Wanderley Garcia de Paula‐Silva, Erika Calvano Kuchler, Raquel Assed Bezerra da Silva, Lea Assed Bezerra da Silva, Jardel Francisco Mazzi‐Chaves, Fabiane Carneiro Lopes‐Olhê, Manoel Damião Sousa‐Neto

**Affiliations:** ^1^ Department of Restorative Dentistry, School of Dentistry of Ribeirão Preto University of São Paulo São Paulo Brazil; ^2^ Faculty of Dentistry, University of Ribeirão Preto São Paulo Brazil; ^3^ Department of Paediatric Dentistry, School of Dentistry of Ribeirão Preto University of São Paulo, Ribeirão Preto São Paulo Brazil; ^4^ Department of Orthodontics, Medical Faculty University Hospital Bonn Bonn Germany

**Keywords:** apical periodontitis, bone metabolism, gene expression, genetic polymorphisms, histopathological analysis

## Abstract

**Aim:**

To evaluate the impact of polymorphisms in *SOCS‐1, TNF‐α* and *RANKL* on gene expression of *RANK*, *RANKL*, *TNFRSF1, SOCS‐1, IL‐10*, *IL‐1β* and *TNF‐α*, and to evaluate the histopathological, immunohistochemical and microbiological aspects of persistent apical periodontitis (PAP) after root canal treatment (RCT) in Brazilian individuals.

**Methodology:**

Patients with pulp necrosis and apical periodontitis at the time of the non‐surgical RCT (NSRCT) were followed up for at least 1 year after NSRCT. In view of the need for surgical intervention (cases assessed with a CBCTPAI score of 4 and 5, with the presence of symptoms), 20 patients were selected for endodontic surgery, which was planned using cone beam computed tomography images. Initially, saliva was collected as a source of genomic DNA, and the individuals were genotyped for *SOCS‐1*, *TNF‐α* and *RANKL* polymorphisms by real‐time PCR. After collecting the biological material, the periapical lesions obtained were subjected to analysis of gene expression levels for *RANK*, *RANKL*, *TNFRSF1, SOCS‐1, IL‐10*, *IL‐1β* and *TNF‐α*, and histopathological evaluation for characterisation and differentiation into periapical granulomas and cysts; immunohistochemical evaluation for SOCS‐1 and IL‐1β protein labeling; and microbiological analysis to identify the microorganisms involved in persistent periapical infection. The relative mRNA expression values of each gene in each group, according with genotypes in different SNPs, were analysed using one‐way analysis of variance followed by Tukey's post‐test or *T*‐test (*α* = 5%).

**Results:**

Different expression values of the genes evaluated were observed according to the genotypes of the polymorphisms evaluated in relation to PAP (*p* < 0.05). Among the cases submitted for histopathological evaluation, 66.7% were diagnosed as periapical granuloma and 33.3% as periapical cyst. Immunohistochemical analysis showed strong positivity for SOCS‐1 and IL‐1β in the lesions classified as periapical cyst, while the lesions diagnosed as periapical granuloma were not labelled. In the microbiological analysis, four different species of bacteria were isolated: *Pseudomonas aeruginosa* , *Staphylococcus epidermidis* , *Enterococcus faecalis* and *Bacillus cereus* .

**Conclusions:**

This exploratory study indicates that genetic polymorphisms can modulate gene expression and protein activity in PAP, shaping the host's inflammatory and reparative response. These findings highlight their potential as biomarkers and establish a basis for future translational studies.

## 1 Introduction

The scientific literature highlights the association between various host genetic components involved in the establishment, progression and repair of apical periodontitis (Aminoshariae and Kulild 2015). Thus, the investigation of the interaction between molecular signals, genetic influence and clinical signs of pulp diseases has been established as a promising research topic in Endodontics. In recent years, interactions between genetic polymorphisms and the different stages of formation and resolution of apical periodontitis have been evidenced in genes linked to inflammation and bone metabolism processes (Petean et al. 2022). Considering that genetic polymorphisms can alter protein synthesis and cellular function (Petean et al. 2022), it is noteworthy that the vast majority of studies investigate only the association of a given polymorphism with disease, but do not demonstrate its actual impact on gene or protein expression, nor on the effective biological response presented by the individual. Thus, genetic polymorphisms can only explain a fraction of the associated risk, since other genetic or epigenetic events (hereditary/acquired characteristics of gene expression and subsequent phenotypic changes that do not include alterations to DNA sequences) can confer additional susceptibility to the disease (Aminoshariae and Kulild 2015; Campos et al. 2015; Fouad et al. 2020; Jakovljevic et al. 2021, 2022).

Regarding the aetiology of apical periodontitis, the release of cytokines and pro‐ and anti‐inflammatory mediators have been shown to be associated with the progression of the periapical lesion and the induction of osteoclastogenesis (Petean et al. 2022), besides the signalising of the body's defence and repair functions (Graves et al. 2011; Dessaune‐Neto et al. 2018; Nikolic et al. 2019). Tumour necrosis factor‐⍺ (TNF‐⍺) is a pro‐inflammatory cytokine that acts as a modulator of the immune response, especially in cases of active lesions with the presence of bone resorption (Garlet 2010), and polymorphisms in the gene that encodes it have already been associated with the progression and persistence of apical periodontitis in humans (Jakovljevic et al. 2020, 2021, 2022; De Castro et al. 2023). Receptor Activator of Nuclear Factor κB Ligand (RANKL) is a soluble mediator and a member of the tumour necrosis factor (TNF) superfamily. When bound to its receptor, RANK (Receptor Activator of Nuclear Factor κB, encoded by TNFRSF11A), this interaction activates downstream signalling pathways, including NF‐κB, thereby promoting osteoclastogenesis. (Graves et al. 2011; Petean et al. 2019). Polymorphisms in *RANKL* have also been associated with the development of persistent periapical lesions after root canal treatment (Petean et al. 2019).

However, the inhibition of pro‐inflammatory cytokines by suppressor of cytokine signalling (SOCS) proteins is an important mechanism for regulating the immune response, including in apical periodontitis (Starr et al. 1997; Alexander and Hilton 2004; Wang et al. 2017). Genetic polymorphisms in *SOCS‐1* have been linked to a shorter period of agranulocytosis, a lower proportion of primitive/immature bone marrow cells, and a higher level of haemoglobin in the blood (Chen et al. 2020), which may be related to the body's ability to repair inflammatory diseases.

In relation to persistent apical periodontitis (PAP) associated with cases of failure after endodontic treatment, the literature shows that the numbers vary between 10% and 15% of cases (Estrela et al. 2014; Lieblich 2020). These lesions indicate failures in the treatment performed and in the host's response to treatment, even if they are asymptomatic (Siqueira and Roças 2008). Failure is dependent on different factors, since the anatomical complexity of the root canal system, in addition to the location of the apical foramen, can make it difficult to completely disinfect the canals (Estrela et al. 2014; Siqueira Junior et al. 2018; Sousa‐Neto et al. 2018). In addition, considering that periapical lesions are a multifactorial disease (Stashenko et al. 1992), the relationship between host resistance and virulence of microorganisms has a direct impact on the development and repair of persistent apical periodontitis (Petean et al. 2022).

When detected, cases of persistent apical periodontitis make reintervention necessary. Within the process of choosing the protocol for reintervention, the literature presents the use of algorithms, in a kind of decision tree, which makes use of data from the patient's symptoms, clinical and imaging examinations for treatment planning, in the search for the best prognosis (Lieblich 2020; Signor et al. 2021; Petean, Gaêta‐Araujo, et al. 2025). Endodontic retreatment is a conservative protocol of first choice, which aims to re‐disinfect the root canal system through biomechanical preparation protocols and the use of intracanal medication, to promote the health and repair of periapical tissues (Del Fabbro et al. 2016; Dioguardi et al. 2022). However, some clinical studies show that although root canal retreatment appears to be more conservative, the removal of posts, root canal repreparation and removal of tooth structure increase the risk of tooth fracture (Lieblich 2020; Stueland et al. 2023). In these cases, surgical treatment can have higher success rates and a better prognosis, as it is a radical approach to infection and allows the use of biomaterials favourable to the regeneration of periapical tissues (Monaghan et al. 2019). It should also be noted that surgical treatment of unsuccessful cases, when indicated, also offers the opportunity to remove tissue for histological examination, allowing further investigation of the disease (Lieblich 2020; Petean, Gaêta‐Araujo, et al. 2025).

Therefore, considering that endodontic failure rates in cases of persistent periapical lesions may be associated with host characteristics, it became opportune to investigate the impact of polymorphisms in *SOCS‐1, TNF‐α* and *RANKL* on the gene expression of *RANK*, *RANKL*, *TNF‐α, TNFRSF1*, *IL‐10*, *IL‐ 1β* and *SOCS‐1*; as well as the histopathological, immunohistochemical and microbiological evaluation of cases of PAP after root canal treatment in Brazilian subjects who underwent surgical intervention. To the best of our knowledge this is the first study to evaluate the real impact of genetic polymorphisms in translation process during the development of PAP. The null hypothesis was that the *SOCS‐1, TNF‐α* and *RANKL* genetic polymorphisms do not impact on gene expression of *RANK*, *RANKL*, *TNF‐α, TNFRSF1*, *IL‐10*, *IL‐ 1β* and *SOCS‐1*.

## 2 Material e Methods

**FIGURE 1:**
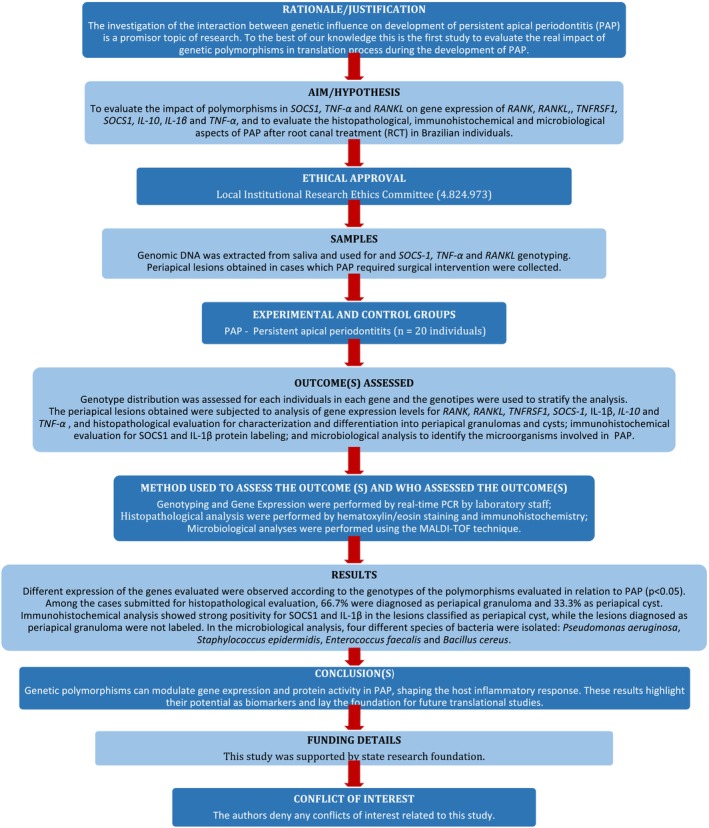
PRILE 2021 flowchart.

The manuscript of this laboratory study was written according to Preferred Reporting Items for Laboratory studies in Endodontology (PRILE) 2021 guidelines (Nagendrababu et al. 2021). Figure 1 is a visual representation of the study design and its outcomes.

### 2.1 Ethical Aspects

This study was reviewed and approved by the local institutional Research Ethics Committee. Written informed consent was obtained with a consent document from all participants, following the ‘Strengthening the Reporting of Genetic Association study (STREGA)’ checklist (Little et al. 2009).

### 2.2 Subjects and Sample Selection

The initial screened cohort comprised 2532 individuals who underwent non‐surgical root canal treatment. Only individuals with pulp necrosis and apical periodontitis at the time of treatment, which had been completed at least 1 year before the follow‐up visit, were considered eligible for this study (Petean et al. 2019; De Castro et al. 2023).

### 2.3 Inclusion and Exclusion Criteria

Regarding the inclusion/exclusion criteria, individuals with at least 1 endodontically treated tooth, with no obvious reason for treatment failure, and with treatment carried out at least 1 year prior to the follow‐up visit were included. Cases were excluded if they presented vertical root fracture, unsatisfactory root canal treatment (filling more than 2 mm from the radiographic apex or gross overfilling and/or presence of voids, inadequate density, unfilled canals or poor condensation), as well as cases with microleakage and/or an unsatisfactory restorative protocol.

In addition, information was collected on the patient's general state of health and habits, and according to the American Society of Anesthesiologists (ASA) physical status classification, only individuals classified as ASA1 and ASA2 status were included (Maloney and Weinberg 2008). Individuals with medical conditions that required the use of systemic bone metabolism modifiers or other assisted drug therapy (i.e., systemic antibiotics, anti‐inflammatories, or hormone therapy) during the previous 6 months prior to the reassessment were excluded.

### 2.4 Clinical and Radiographic Evaluation

At follow‐up visits, the phenotype (treatment outcome) was determined based on radiographic and clinical aspects. At the time of returning for follow‐up, clinical‐radiographic examinations were performed and data on the patient's general state of health and root canal treatment were recorded for all individuals. The immediate post‐operative radiographs were compared with the follow‐up radiographs to compare and determine the periapical health at each time point using the PAI scoring system (Ørstavik et al. 1986). All periapical radiographs were taken using the angle of the bisector without mesial/distal displacement, to avoid distortion. An experienced and calibrated endodontist assessed the radiographs of each individual. In multi‐rooted teeth, the result of the worst root was used to determine the phenotype. Clinical signs or symptoms of periapical disease (i.e., sinus tract, pain and swelling) were also assessed.

Cases of PAP were determined according to previously described criteria (Petean et al. 2019; Petean, Silva‐Sousa, et al. 2025; De Castro et al. 2023), which were: complete treatment for more than a year, with the presence of signs and/or symptoms of disease (e.g., pain, swelling or fistula); absence of complete healing of the periapical lesion, radiographically (radiographically pre‐existing periapical lesion, close to or larger than the initial size, with no signs of progression of the repair); cases in which there are signs of repair and reduction in the size of the lesion, which required longer follow‐up; PAI between scores 3 and 5.

### 2.5 Case Progression and Imaging

From over than 1000 patients who were recalled for clinical/radiographic follow‐up and subsequently subjected to the study's inclusion and exclusion criteria, 172 individuals presented PAP after NSRCT. Most of these patients underwent extended follow‐up, as recommended by the European Society of Endodontology (ESE 2006; Duncan et al. 2023), until complete healing occurred. Others were assessed according to their clinical and radiographic outcomes, as described previously (Petean, Gaêta‐Araujo, et al. 2025), and many were referred for non‐surgical retreatment.

Cases classified as PAI score 5 underwent tomographic evaluation. The CBCT scans were performed following the parameters recommended by the American Association of Endodontics (Low et al. 2008; Venskutonis et al. 2015), using the EAGLE 3D computerised tomography scanner (Dabi Atlante, Ribeirão Preto, SP, Brazil) and the images were acquired using the EAGLE—Advanced program (Dabi Atlante, Ribeirão Preto, SP, Brazil) following the exposure parameters determined for endodontic examinations: high resolution (High Definition Mode—HD) and the smallest possible field of view (FOV), once the area of interest had been determined. Images were reconstructed with On Demand 3D software (Cybermed Inc., Tustin, CA, USA) and assessed using the CBCTPAI scoring system (Estrela et al. 2008).

### 2.6 Decision for Surgical Intervention

Following a predefined decision‐making protocol (Petean, Gaêta‐Araujo, et al. 2025) cases classified as scores 4 and 5 with the presence of expansion/destruction of the cortical bone and complaints of symptoms characteristic of persistent infection were referred for surgical intervention. In total, 20 patients underwent endodontic surgery.

### 2.7 Pre‐Surgical Planning

Previously, the data obtained from the CBCT scans of each patient were used for surgical planning, considering the following aspects: shape, location and spatial position of the lesion; extent of the lesion in relation to the teeth and adjacent anatomical structures; relationship between tooth and periapical lesion; area (mm^2^) of the lesion; density of the lesion and adjacent alveolar bone; degree of bone and root resorption. Aspects of root canal treatment related to the quality of the filling and the persistence of the lesion were also assessed, such as under‐ or over‐filling, leakage of filling material (endodontic sealer or gutta‐percha) and the presence of empty spaces, which were excluded from this study.

### 2.8 Collection of Periapical Lesions

Prior to the endodontic surgeries, all patients underwent oral environment adjustment for restorative and rehabilitative treatment when necessary. The endodontic surgeries were performed by previously calibrated dental surgeons, specialists in endodontics with expertise in the field. The procedures were performed under local anaesthesia and aseptic conditions, with the aid of a surgical microscope (Setzer et al. 2012; Bornstein et al. 2015). The osteotomy, apicectomy, retro‐bonding and suturing procedures were performed according to previous studies (Setzer et al. 2012; Bornstein et al. 2015). The apical fragment of the root and a portion of the tissue, depending on the size of the lesion, were removed, and immediately kept in 10% buffered formalin solution for 48 h for subsequent histological and immunohistochemical processing. Other portions of the periapical lesions, where possible according to the size of the lesion, were frozen in a freezer (−20°C) for subsequent PCR analysis.

### 2.9 CBCT Follow‐Up of Patients Undergoing Endodontic Surgery

Patients who had undergone endodontic surgery were referred for a new CBCT scan to follow up the case 6 months after the surgical intervention, following the same parameters adopted previously using the EAGLE 3D CT scanner (Dabi Atlante, Ribeirão Preto, SP, Brazil) at FORP/USP. The images were then reconstructed using On Demand 3D program (Cybermed Inc., Tustin, CA, USA).

### 2.10 Collection of Biological Material and DNA Extraction

Before surgical intervention, saliva samples were collected as a source of genomic DNA. The samples were collected by swishing with 5 mL of 5% saline solution for 1 min, and the entire volume of the swish was placed in 15 mL falcon tubes (Corning Inc., Corning, NY, USA). Genomic DNA for genotyping analysis was extracted from oral cells isolated from saliva, as previously established (Küchler et al. 2012). The quantity and purity of the DNA were determined by spectrophotometry (Nanodrop 1000; Thermo Scientific, Wilmington, DE, USA).

### 2.11 Selection of Genetic Polymorphisms and Genotyping by PCR

Polymorphisms in the *SOCS‐1, TNF‐α* and *RANKL* genes were used to assess genetic variations and selected based on previous association studies (Menezes, Garlet, Trombone, et al. 2008; Fukushima et al. 2010; Bek et al. 2016; Wang et al. 2017; Crommelin et al. 2020; Jakovljevic et al. 2022; De Castro et al. 2023), which are either classified as potentially functional or influence gene expression (Table 1).

**TABLE 1:** Description of the genes and genetic polymorphisms selected for evaluation in this study.

Gene	Polymorphism	Base changes^a^	Functional consequence	Global MAF
*SOCS‐1*	*rs243327*	ACT**[A/G]**GCC	Intron variant	0.3910
*TNF‐α*	*rs1800629*	ATG[**A/G**]GGA	Intron variant	0.1553
*RANKL*	*rs1054016*	TTT[**G/T**]TTT	UTR variant	0.3329

^a^ Allele in bold indicates the ancestral allele. Global MAF is the frequency of the smallest allele in the global population (Ethnic division: Caucasian). *Source:* http://www.ncbi.nlm.nih.gov/snp/; http://genome.ucsc.edu/; http://www.thermofisher.com/.

Genotyping of the selected polymorphisms [*SOCS‐1* rs243327 (Applied Biosystems Assay ID: C___3189840_10), *TNF‐α* rs1800629 (Applied Biosystems Assay ID: C___7514879_10), and *RANKL* rs1054016 (Applied Biosystems Assay ID: C___7444426_10)] was performed by PCR using the TaqMan system (Ranade et al. 2001). The real‐ time PCR reactions were performed in duplicate, in a total volume of 5.15 μL (6 ng DNA/reaction, 2 μL Taqman PCR master mix, 0.15 SNP assay; Applied Biosystems, Foster City, CA). Thermal cycling was performed starting with a heating cycle of 95°C for 10 min, followed by 40 amplification cycles of 92°C for 15 s and 60°C for 1 min (StepOnePlus Real‐Time PCR System; Thermo Fisher Scientific, Massachusetts, USA). Genotyping was successfully performed for all surgical cases, with a 100% sample yield for the selected polymorphisms.

### 2.12 Gene Expression Analysis by PCR

The mediators *RANK* (*TNFSF11*, Applied Biosystems Assay ID: Hs00243522_m1), *RANKL* (*TNFRSF11A*, Applied Biosystems Assay ID: Hs00921369_m1), *TNFRSF1* (Applied Biosystems Assay ID: Hs00900358_m1), *SOCS‐1* (Applied Biosystems Assay ID: Hs00705164_s1), IL‐1β (Applied Biosystems Assay ID: Hs01555410_m1), *IL‐10* (Applied Biosystems Assay ID: Hs00961622_m1) and TNF (Applied Biosystems Assay ID: Hs00174128_m1) were assessed. *ACTB* (Applied Biosystems Assay ID: Hs99999903_m1) and *GAPDH* (Applied Biosystems Assay ID: Hs02786624_g1) was used as reference gene. The mirVanaTM miRNA Isolation kit (Ambion/Life TechnologiesTM) was used to extract total RNA from periapical lesions. Nucleic acid quantification was performed using a NanoDropTMOne spectrophotometer (Thermo Fisher Scientific, Massachusetts, USA). Complementary DNA (cDNA) was synthesised using 1 μg of total RNA and the High‐Capacity kit (Applied Biosystems, Foster City, California, USA). The samples were submitted to the RT‐PCR StepOnePlusTM Real‐Time PCR System (Thermo Fisher Scientific, Massachusetts, USA) for the thermocycling program recommended by the manufacturer of the reverse transcription kit used. The cDNA was then used for the PCR quantification assay (qPCR) in duplicates to analyse gene expression levels with the TaqManTM system (Applied Biosystems, Foster City, California, USA). The relative expression was calculated using the {2‐Δ(ΔCt)} method. RNA extraction, reverse transcription, and qPCR analysis were successfully completed for all surgical specimens, achieving a 100% yield across the panel of target genes.

### 2.13 Histopathological Evaluation

The samples were fixed in 10% buffered formalin and subjected to conventional histological processing and embedded in paraffin blocks. Next, 5 μm histological sections were obtained using a hand‐held microtome and subjected to haematoxylin and eosin (H&E) staining. The H&E‐stained samples were used to determine the final diagnosis of the persistent periapical lesions. This diagnosis was based on the histopathological characteristics observed through photomicroscope analysis (Zeiss Axio Imager, Carl Zeiss AG Light Microscopy, Gottingen, Germany) under conventional light. For the final diagnosis, the following were considered: the presence of dense connective tissue associated with a diffuse inflammatory infiltrate (Periapical Granuloma) or the presence of connective tissue associated with the presence of stratified epithelium, which may or may not be lining a cystic cavity, depending on the integrity of the sample, which characterises a cystic lesion and is classified as a Periapical Cyst. Overall, approximately 60% of the specimens yielded material of adequate quality and quantity to be processed successfully.

### 2.14 Immunohistochemical Evaluation

To analyse protein expression by immunohistochemistry, the samples were cut into 3 μm histological sections, deparaffinised and rehydrated. Antigen retrieval was performed using citrate buffer solution in a water bath at 95°C–99°C for 5 min, followed by peroxidase blocking and incubation with bovine serum albumin (5% BSA) for 1 h at room temperature. Subsequently, primary antibodies were applied at final dilutions of 1:100 (SOCS‐1) and 1:200 (IL‐1β), and the slides were incubated overnight at 4°C in a humid chamber. HRP‐conjugated secondary antibodies were used, and immunoreactivity was visualised with DAB. After PBS washing, counterstaining was performed with haematoxylin for 5 min. Finally, samples were dehydrated and mounted for microscopic analysis (Zeiss Axio Imager, Carl Zeiss AG Light Microscopy, Gottingen, Germany) under conventional light and images captured at 20X magnification to assess the presence or absence of immunolabelling of the selected proteins as well as their location.

### 2.15 Microbiological Assessment

Microbiological analyses were performed to identify the microorganisms involved in the periapical infection. Biological material was collected during endodontic surgery using sterile paper points when the periapical lesion was excised, to collect biofilm material from the apical zone. The collections were made in duplicate and one point was transferred to a test tube containing 5 mL of Brain Heart Infusion Broth (BHI) and the other cone to a tube containing an equal volume of Thioglycolate Broth (Acumedia, USA), to allow the viability of aerobic and anaerobic bacteria, respectively (Murray et al. 2007). The MALDI‐TOF (Matrix‐assisted laser desorption ionisation‐time of flight) technique was used to identify the isolates, analysed by Maldi Biotyper Compass 1.4 and Flex Control operating systems (Bruker Daltonics, Bremen, Germany). The spectral peaks (wavelength 260–337 nm) were compared with a library provided by Bruker Daltonics Inc. Bremer, Germany. Score values ≥ 1.7 were considered ered for identification at genus and species level (McElvania Tekippe et al. 2013). Overall, approximately 80% of the specimens yielded material of adequate quality and quantity for successful processing.

### 2.16 Statistical Analysis

For gene expression analyses, relative quantification was performed using the 2^−ΔΔCt method. Fold‐change values were obtained by normalising each target gene to the reference genes and comparing expression levels across genotypes. The most common homozygous genotype was used as the reference group, serving as the baseline for fold‐change calculations. Relative mRNA expression values were compared using one‐way analysis of variance (ANOVA) followed by Tukey's post hoc test or Student's *t*‐test (*α* = 0.05), after verification of the assumptions of normality and homogeneity of variances. Post hoc estimates of the power achieved by the analyses were calculated in G*POWER 3.1.9.6, using an alpha level of 0.05 and the observed effect sizes for each comparison.

## 3 Results

### 3.1 Analysis of Genetic Polymorphisms

Genotyping was successfully performed for all surgical cases, with a 100% sample yield (*n* = 20) for the selected polymorphisms. The distribution of genotypes and corresponding allele frequencies for *SOCS‐1* (rs243327), *TNF‐α* (rs1800629), and *RANKL* (rs1054016) are summarised in Table 2. For the rs243327 polymorphism in *SOCS‐1*, 7 individuals (35%) presented the GG genotype (common homozygote), 9 (45%) the AG genotype, and 4 (20%) the AA genotype. For the rs1800629 polymorphism in *TNF‐α*, 15 individuals (75%) carried the GG genotype (common homozygote) and 5 (25%) the AG genotype. For the rs1054016 polymorphism in *RANKL*, 10 individuals (50%) presented the GG genotype (common homozygote), 7 (35%) the GT genotype, and 3 (15%) the TT genotype.

**TABLE 2:** Genotype and allele frequencies of *SOCS‐1* (rs243327), *TNF‐α* (rs1800629), and *RANKL* (rs1054016) polymorphisms in patients who underwent surgical management of PAP.

Gene	Polymorphism	Genotype	*n* (%)	Allele frequency (%)
*SOCS‐1*	*rs243327*	GG	7 (35.0)	G: 57.5 A: 42.5
		AG	9 (45.0)	
		AA	4 (20.0)	
*TNF‐α*	*rs1800629*	GG	15 (75.0)	G: 87.5 A: 12.5
		AG	5 (25.0)	
		AA	0 (0.0)	
*RANKL*	*rs1054016*	GG	10 (50.0)	G: 67.5 T: 32.5
		GT	7 (35.0)	
		TT	3 (15.0)	

### 3.2 Analysis of Gene Expression in Periapical Lesions Obtained During Endodontic Surgery

**FIGURE 2:**
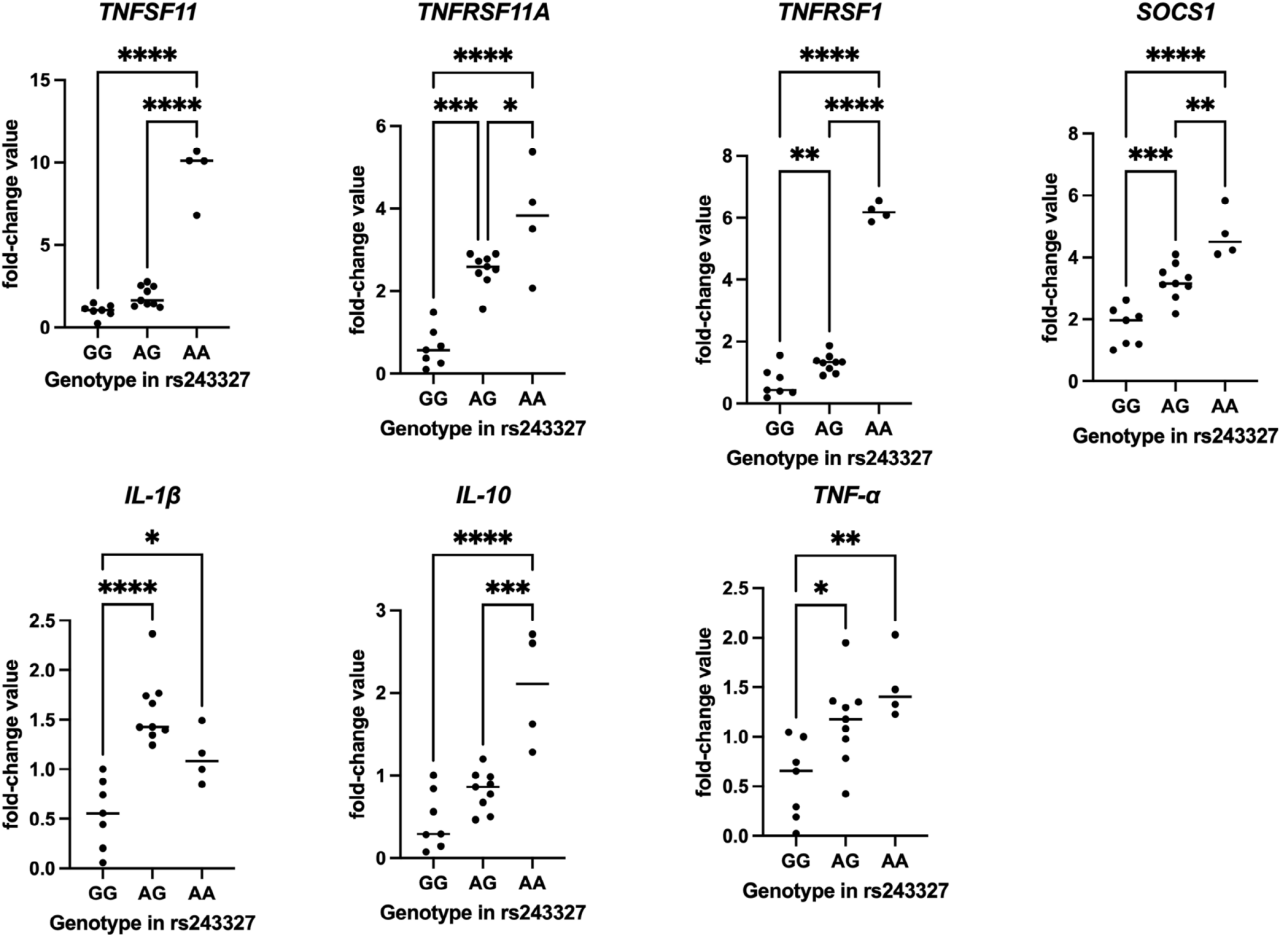
Relative mRNA expression values (fold change) of target genes according to rs243327 genotypes in *SOCS‐1*. Each dot represents an individual sample, normalised to reference genes. Horizontal bars indicate group means. Asterisks denote statistically significant differences between groups.

Table 3 presents the relative expression values (mean ± standard deviation) of the *RANKL (TNFSF11), RANK (TNFRSF11A), TNFRSF1, SOCS‐1, IL‐1β, IL‐10*, and *TNF‐α* genes, distributed according to the genotypes observed for the rs243327 polymorphism in *SOCS‐1*. Higher expression levels of *RANK (TNFRSF11A), RANKL (TNFSF11), TNF‐α, TNFRSF1, IL‐10*, and *SOCS‐1* were observed in AA individuals, whereas AG individuals showed intermediate expression levels and GG individuals showed the lowest expression levels (*p* < 0.05). For *IL‐1β*, expression was highest in AG individuals, intermediate in AA individuals, and lowest in GG individuals (*p* < 0.05). Figure 2 presents the individual fold‐change values of all 20 patients, according to their genotype distribution for the rs243327 polymorphism in *SOCS‐1*.

**TABLE 3:** Relative expression of the *TNFSF11, TNFRSF11A, TNFRSF1, SOCS‐1, IL‐1β, IL10* and *TNF‐α* genes among individuals of different genotypes observed in the *SOCS1* polymorphism (rs243327).

Gene	Genotype and relative expression mean (SD)				
	GG	AG	AA	*p* ^a^	Power estimate
*TNFSF11*	1.01 (0.40) B	1.89 (0.60) B	9.42 (1.77) A	**< 0.0001**	0.96
*TNFRSF11A*	0.63 (0.47) C	2.52 (0.41) A	3.77 (1.37) A	**< 0.0001**	0.97
*TNFRSF1*	0.68 (0.47) C	1.30 (0.29) B	6.19 (0.29) A	**< 0.0001**	0.99
*SOCS‐1*	1.77 (0.62) C	3.22 (0.57) B	4.73 (0.78) A	**< 0.0001**	0.95
*IL‐1β*	0.55 (0.34) B	1.59 (0.34) A	1.12 (0.27) A	**< 0.0001**	0.98
*IL10*	0.45 (0.35) B	0.81 (0.24) B	2.05 (0.70) A	**< 0.0001**	0.96
*TNF‐α*	0.56 (0.40) B	1.15 (0.42) B	1.51 (0.35) A	**0.0037**	0.95

*Note:* (SD) means standard deviation. Fold‐change values are expressed relative to the most common homozygous genotype (GG).

^a^ One‐way ANOVA. Numbers in bold and different letters on the same line represent a statistically significant difference (*p* ≤ 0.05).

**FIGURE 3:**
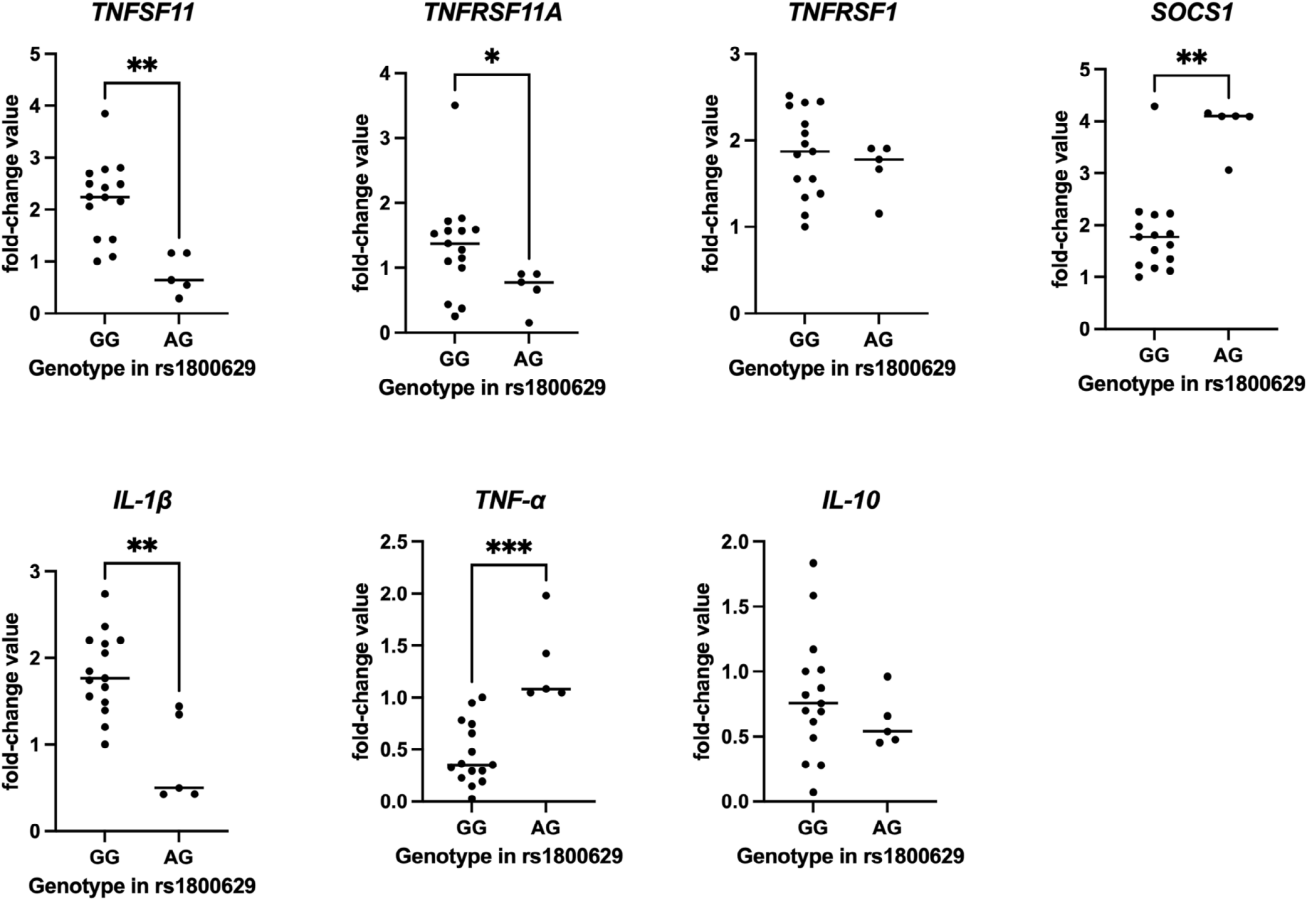
Relative mRNA expression values (fold change) of target genes according to rs1800629 genotypes in *TNF‐α*. Each dot represents an individual sample, normalised to reference genes. Horizontal bars indicate group means. Asterisks denote statistically significant differences between groups.

Table 4 presents the relative expression values (mean ± standard deviation) of the same genes, distributed according to the genotypes observed for the rs1800629 polymorphism in *TNF‐α*. Higher expression levels of *RANK, RANKL* and *IL‐1β* were observed in GG individuals, whereas AG individuals showed lower expression levels (*p* < 0.05). For *SOCS‐1* and *TNF‐α*, higher expression levels were observed in AG individuals and lower expression levels in GG individuals (*p* < 0.05). For *TNFRSF1* and IL‐10, no statistically significant differences were observed between the evaluated groups (*p* > 0.05). No AA genotype was identified among the individuals analysed. Figure 3 presents the individual fold‐change values of all 20 patients, according to their genotype distribution for the rs1800629 polymorphism in *TNF‐α*.

**TABLE 4:** Relative expression of the *TNFSF11, TNFRSF11A, TNFRSF1, SOCS‐1, IL‐1β, IL10* and *TNF‐α* genes among individuals of different genotypes observed in the *TNF* polymorphism (rs1800629).

Gene	Genotype and relative expression mean (SD)				
	GG	AG	AA	*p* ^a^	Power estimate
*TNFSF11*	2.21 (0.74) A	0.75 (0.38) B	—	**0.0014**	0.95
*TNFRSF11A*	1.34 (0.68) A	0.68 (0.31) B	—	**0.0254**	0.95
*TNFRSF1*	1.84 (0.50) A	1.68 (0.31) A	—	0.5531	0.95
*SOCS‐1*	1.82 (0.79) B	3.90 (0.47) A	—	**0.0025**	0.97
*IL‐1β*	1.82 (0.46) A	0.82 (0.51) B	—	**0.0025**	0.96
*IL10*	0.81 (0.47) A	0.61 (0.20) A	—	0.3056	0.95
*TNF‐α*	0.45 (0.29) A	1.31 (0.40) B	—	**0.0001**	0.95

*Note:* (SD) means standard deviation. Fold‐change values are expressed relative to the most common homozygous genotype (GG).

^a^ *t*‐test. Numbers in bold and different letters on the same line represent a statistically significant difference (*p* ≤ 0.05).

**FIGURE 4:**
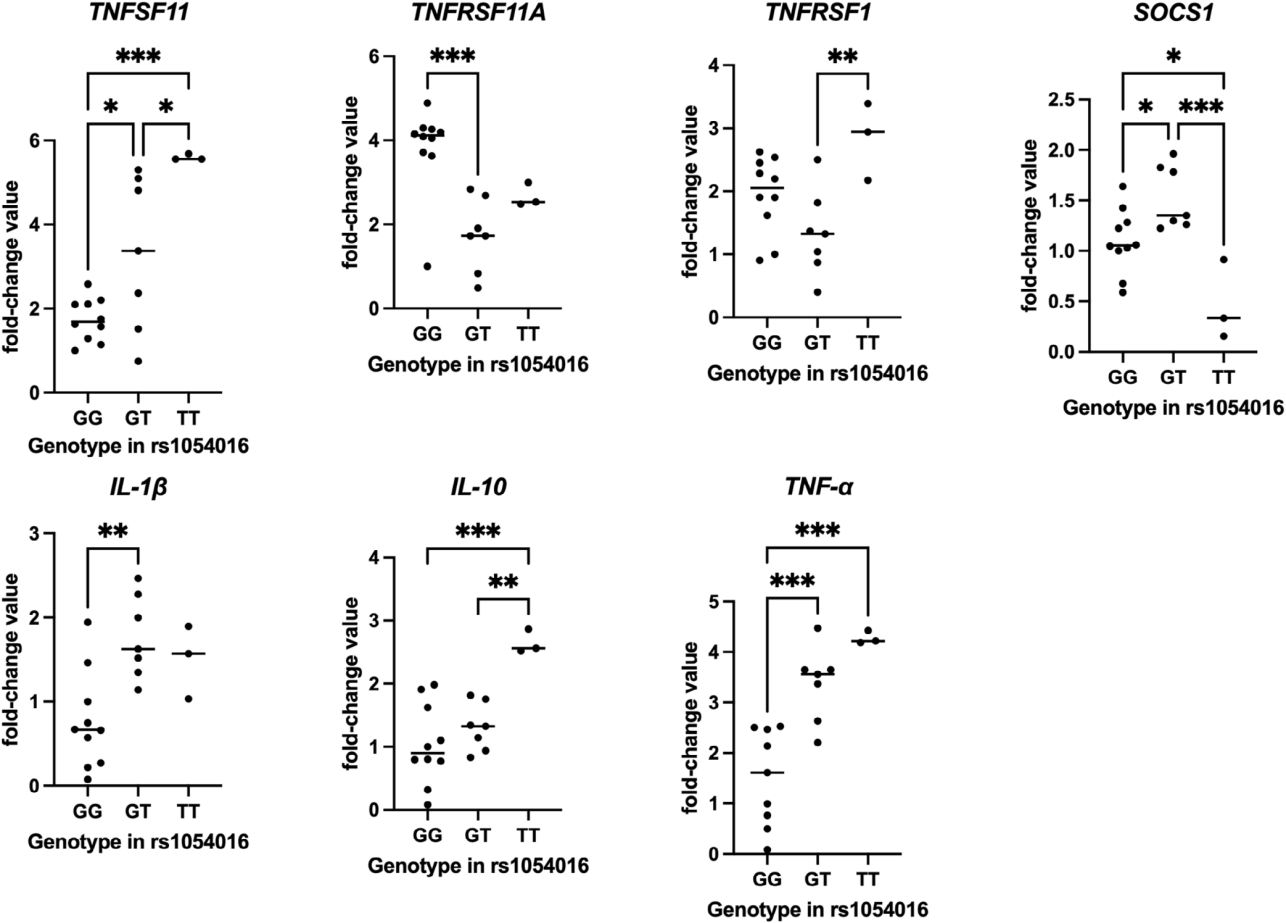
Relative mRNA expression values (fold change) of target genes according to rs1054016 genotypes in *RANKL*. Each dot represents an individual sample, normalised to reference genes. Horizontal bars indicate group means. Asterisks denote statistically significant differences between groups.

Table 5 presents the relative expression values (mean ± standard deviation) of the same genes, distributed according to the genotypes observed for the rs1054016 polymorphism in *RANKL*. Higher expression levels of *RANKL (TNFSF11)* were observed in TT individuals, whereas intermediate expression levels were found in GT individuals, and the lowest expression levels were detected in GG individuals (*p* < 0.05). For *RANK (TNFRSF11A)*, expression was highest in GG individuals, intermediate in TT individuals, and lowest in GT individuals (*p* < 0.05). Higher expression levels of *TNFRSF1* were observed in TT individuals, intermediate in GG individuals, and lowest in GT individuals (*p* < 0.05). For *SOCS‐1*, GG and GT individuals presented similar expression levels (*p* > 0.05), whereas TT individuals showed significantly lower values (*p* < 0.05). *IL‐1β* expression was highest in GT and TT individuals (no significant difference between them, *p* > 0.05), both of which expressed higher levels compared with GG individuals (*p* < 0.05). For *IL‐10*, expression was highest in TT individuals (*p* < 0.05), whereas GG and GT individuals presented similar expression patterns (*p* > 0.05). Finally, for *TNF*, expression levels followed the order TT>GT > GG (*p* < 0.05). Figure 4 presents the individual fold‐change values of all 20 patients, according to their genotype distribution for the rs1054016 polymorphism in *RANKL*.

**TABLE 5:** Relative expression of the *TNFSF11, TNFRSF11A, TNFRSF1, SOCS‐1, IL‐1β, IL10* AND *TNF‐α* genes among individuals of different genotypes observed in the *RANKL* polymorphism (rs1054016).

Gene	Genotype and relative expression mean (SD)				
	GG	GT	TT	*p* ^a^	Power estimate
*TNFSF11*	1.73 (0.50) B	3.31 (1.83) AB	5.59 (0.07) A	**0.0003**	0.95
*TNFRSF11A*	3.82 (1.05) A	1.74 (0.86) B	2.67 (0.28) AB	**0.0011**	0.96
*TNFRSF1*	1.94 (0.60) AB	1.33 (0.67) B	2.83 (0.61) A	**0.0104**	0.95
*SOCS‐1*	1.09 (0.31) A	1.53 (0.31) AB	0.46 (0.39) B	**0.0007**	0.96
*IL‐1β*	0.76 (0.57) B	1.76 (0.49) A	1.50 (0.43) A	**0.0040**	0.95
*IL10*	1.03 (0.63) B	1.30 (0.37) B	2.64 (0.19) A	**0.0008**	0.95
*TNF‐α*	1.51 (0.95) B	3.36 (0.74) A	4.27 (0.13) A	**< 0.0001**	0.98

*Note:* (SD) means standard deviation. Fold‐change values are expressed relative to the most common homozygous genotype (GG).

^a^ One‐way ANOVA. Numbers in bold and different letters on the same line represent a statistically significant difference (*p* ≤ 0.05).

### 3.3 Histopathological Evaluation

**FIGURE 5:**
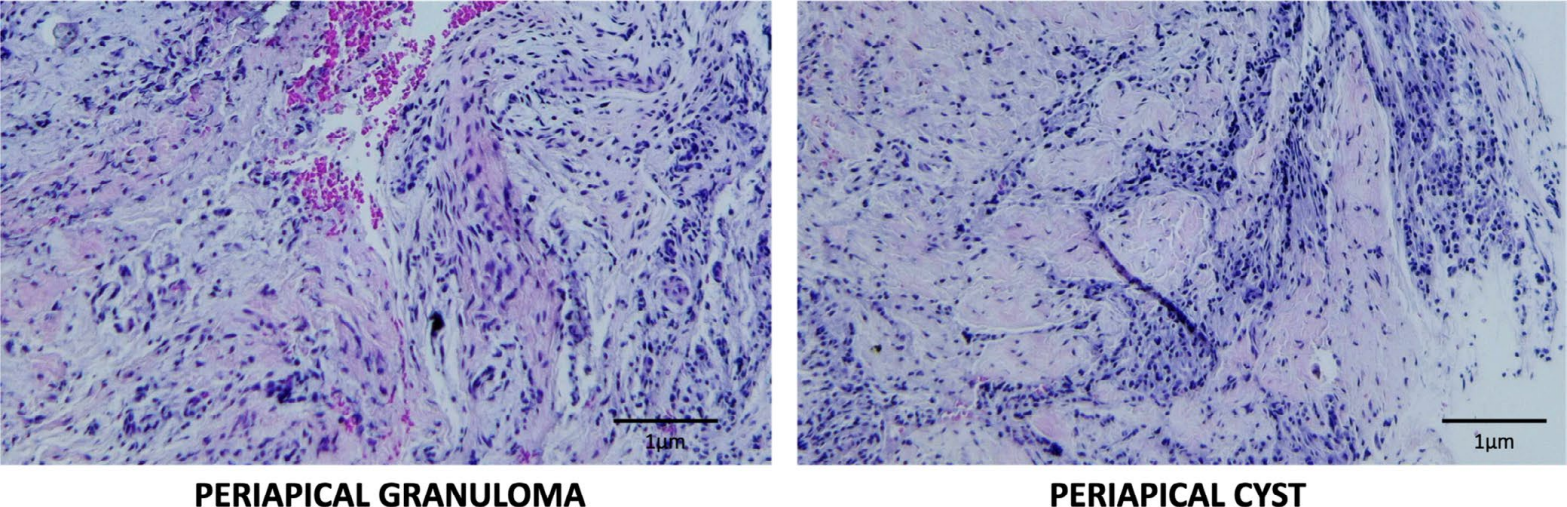
Photomicrographs of histological sections of representative samples of apical lesions, stained in H&E (20×).

The histopathological analysis of the lesions, performed by taking photomicrographs of the histological sections stained with haematoxylin and eosin (Figure 5) showed that among the 12 cases submitted for histopathological evaluation, 8 (66.7%) were diagnosed as periapical granulomas, with the presence of diffuse inflammatory infiltrate and neovascularisation, while 4 (33.3%) were diagnosed as periapical cysts, which also showed the presence of inflammatory infiltrate, but associated with stratified cystic epithelium of odontogenic origin.

### 3.4 Immunohistochemical Evaluation

**FIGURE 6:**
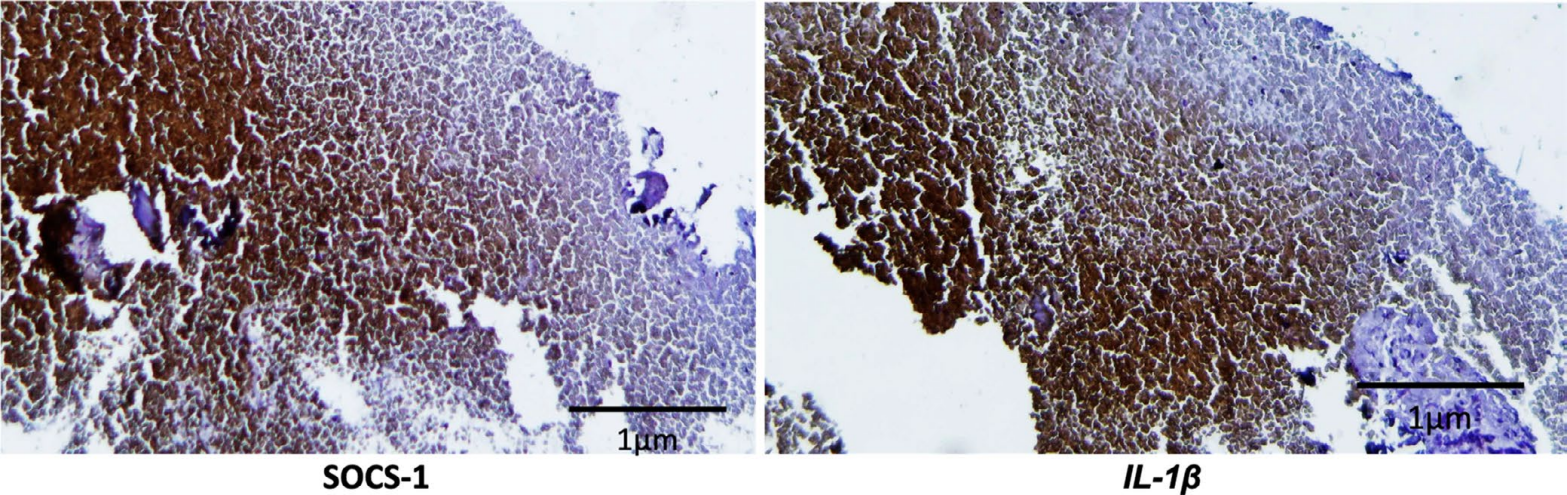
Photomicrographs of histological sections of representative samples of periapical cyst lesions, submitted to immunohistochemistry (20×).

**FIGURE 7:**
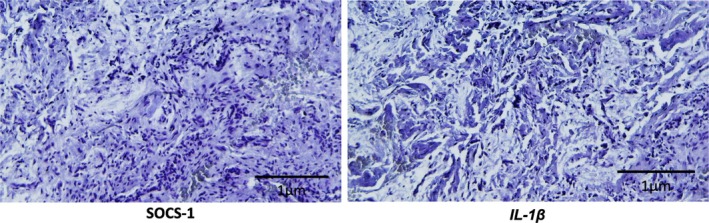
Photomicrographs of histological sections of representative samples of periapical granuloma lesions, submitted to immunohistochemistry (20×).

The immunohistochemical analyses showed strong positivity of SOCS‐1 and IL‐1β for the lesions classified as periapical cysts (Figure 6), while the lesions diagnosed as periapical granulomas showed no expression of the selected proteins (Figure 7).

### 3.5 Microbiological Assessment

Microbiological analysis revealed that four different species of bacteria were isolated from the samples collected in this study. These were identified as *Pseudomonas aeruginosa (*present in 44.4% of the samples), *Staphylococcus epidermidis* (present in 33.3% of the samples), *Enterococcus faecalis (present in* 11.2% of the samples) and *Bacillus cereus* (present in 11.1% of the samples).

## 4 Discussion

Persistent apical periodontitis should be treated with an approach to the particularities of the microbiology, the quality of the treatment and the host's response to the proposed treatment (Küchler et al. 2018; Petean et al. 2022). Thus, considering that genetic polymorphisms can alter the synthesis of a protein and cellular function (Fouad et al. 2020; Petean et al. 2022), this study aimed to investigate the real impact of genetic polymorphisms on the development of persistent apical periodontitis. According to the results obtained, genetic polymorphisms in the *SOCS‐1, TNF‐α* and *RANKL* genes influenced the expression of mediators linked to inflammation and bone metabolism, rejecting the null hypothesis of the present study.

Based on the samples evaluated in this study, it was observed that in the distribution of *SOCS‐1* genotypes (rs243327) in individuals undergoing endodontic surgery to collect periapical lesions, AA individuals showed higher expression of *RANK, RANKL, TNFRSF1, SOCS‐1, IL‐10* and *TNF‐α*, AG individuals showed higher expression of *IL‐1β*, while GG individuals showed lower expression for all the genes evaluated. In view of this, it should be noted that *SOCS‐1* is activated by the presence of interferon, tumour necrosis factor, IL‐ 6, or by exposure to bacterial lipopolysaccharide, and its action occurs by inhibiting the expression of the same interferon, tumour necrosis factor and IL‐6 (Starr et al. 1997; Morita et al. 2000; Alexander 2002; Nakagawa et al. 2002; Alexander and Hilton 2004; Guimarães et al. 2013). *SOCS‐3*, on the other hand, generally acts on the expression of *IL‐1, IL‐6, IL‐10* and *interferon‐gamma* (Alexander 2002; Alexander and Hilton 2004; Rakesh and Agrawal 2005; Dessaune‐Neto et al. 2018), and has been found in periapical granulomas, together with the expression of IL‐10 (Menezes, Garlet, Trombone, et al. 2008). In addition, it is known that SOCS‐3 expression is induced by pro‐inflammatory cytokines and this condition inhibits the secretion of chemokines induced by *IL‐1β* or IL‐6, thus, SOCS‐3 protein expression in humans plays important negative feedback, suppressing the progression of periapical disease (Fukushima et al. 2010). Therefore, there is a compensatory mechanism that may justify the results of the expressions of *SOCS‐1* and the other genes evaluated.

In relation to the rs1800629 polymorphism in *TNF‐*⍺, it was observed that GG individuals showed greater expression of *RANK, RANKL* and *IL‐1β*, while AG individuals showed greater expression of the *SOCS‐1* and *TNF‐*⍺ genes, while the AA genotype was not observed in any of the individuals undergoing endodontic surgery. The scientific literature shows that the A allele has a protective effect on the development of persistent apical periodontitis (De Castro et al. 2023; Petean, Silva‐Sousa, et al. 2025). On the other hand, in relation to the effect of variations in *TNF‐*⍺, a previous study demonstrated that substitution by the A allele has been related to higher levels of TNF‐⍺ (Louis et al. 1998), as observed in the present study for the gene expression of *SOCS‐1* and *TNF‐*⍺. The lack of this allele, on the other hand, led to higher expressions of *RANK, RANKL* and *IL‐1β* in GG individuals, which were associated with a higher risk of developing PAP (De Castro et al. 2023; Petean, Silva‐Sousa, et al. 2025), which can be attributed to the catabolic effects of these genes on bone metabolism (Menezes, Garlet, Trombone, et al. 2008; Nikolic et al. 2019).

Regarding the rs1054016 polymorphism in *RANKL*, the analysis of gene expression in samples of human periapical lesions showed higher values for *RANKL, TNFRSF1, IL‐10* and *TNF‐*⍺ in TT individuals. Although the scientific literature shows the protective effect of the T allele in *RANKL* polymorphisms (Petean et al. 2019), the data obtained in the present study should be interpreted in the context of the suppressive activity of SOCS‐1, the anti‐inflammatory role of IL‐10, the balance between RANKL and OPG, as well as the translational capacity of these genes and their influence on protein activity (Alexander 2002; Alexander and Hilton 2004; Menezes, Garlet, Letra, et al. 2008; Menezes, Garlet, Trombone, et al. 2008; Siqueira Jr et al. 2011; Trombone et al. 2016; Petean et al. 2019; Cavalla et al. 2021). In addition, the interaction between *RANKL* and *TNF* polymorphisms, which have been strongly associated with persistent apical periodontitis (Petean, Silva‐Sousa, et al. 2025), must also be considered.

In the histopathological analysis, this study found a higher prevalence of periapical granulomas compared to periapical cysts. The literature presents controversial data regarding the prevalence ratio between these lesions: while a review reported a predominance of cysts, especially in cases of persistent apical periodontitis after endodontic treatment (Couto et al. 2021), other studies indicate that granulomas are more frequent and that cysts may evolve from granulomas, highlighting the importance of considering the timing of surgical intervention (Enriquez et al. 2015; Sullivan et al. 2016; Lieblich 2020; Petean, Gaêta‐Araujo, et al. 2025).

Considering the evaluation of chronic lesions, the entire stromal compartment was examined, including epithelial lining, fibrous tissue, and inflammatory infiltrate. Immunohistochemical analysis revealed that periapical cysts exhibited stronger immunoreactivity for SOCS‐1 and IL‐1β, with staining predominantly localised to the epithelial lining and, to a lesser extent, within the inflammatory infiltrate, whereas granulomas consistently lacked detectable expression of these proteins. Although previous studies reported increased SOCS expression in granulomas (Menezes, Garlet, Trombone, et al. 2008), this was not observed here, a discrepancy that may be explained by their dense collagenous stroma and reduced inflammatory profile, which could restrict antigen accessibility and cellular activation. This supports the concept that granulomas frequently represent quiescent tissue with limited reparative capacity, behaving more like collagenous scars than active inflammatory entities (Lee et al. 2021; Boonkasemsanti et al. 2025). From a biological perspective, this reduced inflammatory activity and predominance of fibrotic stroma in granulomas may delay bone regeneration and repair, contributing to lesion persistence even after adequate endodontic treatment. Clinically, differentiating between highly inflamed lesions, which may resolve more readily, and fibrotic ones, which tend to show a refractory healing response, is of major relevance. Future studies combining molecular markers, extracellular matrix characterisation, and longitudinal clinical follow‐up could help to clarify the heterogeneous behaviour of these lesions. Conversely, the expression of SOCS proteins in cysts may reflect the activity of undifferentiated mesenchymal cells of odontogenic origin, contributing not only to lesion persistence but also to their recurrence potential. In this context, SOCS proteins may exert a dual role by modulating pro‐inflammatory cytokine activity and influencing the biological behaviour of periapical lesions, particularly in relation to local inflammation and alveolar bone resorption (Menezes, Garlet, Trombone, et al. 2008; Petean et al. 2022).

The development of PAP is essentially linked to bacterial infection, and the literature has consistently demonstrated that genera such as *Fusobacterium*, *Parvimonas*, *Prevotella*, *Porphyromonas*, *Dialister*, *Streptococcus* and *Treponema* are frequently associated with periapical lesions, often detected as coaggregated communities within biofilms (Siqueira Jr et al. 2014; Horiuchi et al. 2020). In most cases, intraradicular infection is the main etiological factor responsible for the formation of granulomas and cysts (Ricucci and Siqueira Jr. 2010). This well‐established microbial background provides a useful framework for interpreting the findings of the present study.

In our samples, however, the microbiological analysis revealed the presence of *Staphylococcus epidermidis* (gram‐positive), *P. aeruginosa* (gram‐negative), *E. faecalis* (gram‐positive), and *B. cereus* (gram‐positive). These isolates, although not the genera most frequently reported in classic endodontic infections, illustrate the heterogeneity of microorganisms that may persist in periapical lesions and highlight the importance of broad‐spectrum and complementary disinfection strategies, including irrigants and intracanal medication, to reduce intra‐ and extraradicular bacterial content (Siqueira Jr and Rôças 2022). The purpose of this microbiological assessment was twofold: first, to confirm the viability and persistence of contaminating bacteria during the experimental period; and second, to correlate the microbial profile with the host inflammatory response, thereby providing a more comprehensive characterisation of the experimental model.

Of particular note, *P. aeruginosa* was the most prevalent species in our study (44.4%). As a gram‐negative bacterium, it contains highly immunogenic lipopolysaccharides (LPS) that strongly activate TLR4/NF‐κB signalling, inducing the release of pro‐inflammatory cytokines such as IL‐1β, IL‐6 and TNF‐α (Sadikot et al. 2005; De Soyza et al. 2013). The predominance of this microorganism, therefore, provides a plausible link to the molecular findings of elevated inflammatory mediator expression. Furthermore, the coexistence of gram‐positive ( *S. epidermidis* , *E. faecalis* , *B. cereus* ) and gram‐negative ( *P. aeruginosa* ) bacteria may synergistically stimulate both TLR2‐ and TLR4‐mediated pathways, amplifying cytokine production and aggravating periapical inflammation (Kristian et al. 2005; Bachtiar and Bachtiar 2017; Rider et al. 2016). Taken together, these findings reinforce the biological plausibility of the model and highlight the importance of associating microbiological, molecular and immunological analyses for a comprehensive understanding of the mechanisms underlying PAP.

The present study has an important limitation that must be acknowledged: the relatively small number of surgical cases available for molecular, histopathological, immunohistochemical, and microbiological analyses. This reflects the fact that only a very small fraction of the large screened population met the strict eligibility criteria for surgical intervention, which constituted the sole source of biological material. It is also important to note that the surgical indication rate observed in this study (< 2%) was substantially lower than the 10%–15% reported in histopathological series (Kim and Kratchman 2006). This difference reflects the specific methodological design and clinical characteristics of our cohort. The denominator used in our analysis encompassed the entire screened population of patients who underwent nonsurgical root canal treatment and were followed up clinically and radiographically, rather than a preselected failure population. Consequently, only a small subset met the strict clinical and radiographic criteria for surgical intervention, ensuring high internal validity but inevitably limiting sample size. Although this inherently limits the generalisability of the findings and the statistical power typically expected in genetic association studies, a post hoc power analysis confirmed that most comparisons achieved power values above 90%. Importantly, the rigorous inclusion criteria ensured the homogeneity, reliability, and clinical relevance of the selected samples, mitigating potential sources of bias. Accordingly, this study should be interpreted as exploratory and hypothesis‐generating, providing preliminary but biologically plausible evidence on how genetic polymorphisms may influence gene expression, protein activity, and host–pathogen interactions in PAP. To the best of our knowledge, this is the first translational study to simultaneously integrate genetic polymorphisms, gene expression, histopathological features, protein immunolabelling, and microbial profiling in PAP. These findings, while requiring validation in larger multicenter cohorts, offer meaningful insights into the molecular and cellular mechanisms underlying disease persistence and provide a robust basis for future translational research in Endodontics.

It should also be noted that, in the evaluation of the success of nonsurgical endodontic treatment, especially in cases of apical periodontitis, the follow‐up period plays a critical role in determining the host's biological response to therapy (Petean et al. 2019; Duncan et al. 2023). The American Association of Endodontists recommends that cases be monitored for up to 5 years after nonsurgical root canal treatment before a persistent periapical lesion can be classified as post‐treatment disease (Penesis et al. 2008). The European Society of Endodontology (ESE) previously recommended in its 2006 Quality Guidelines that both nonsurgical and surgical treatments be evaluated at least 1 year after completion and, when necessary, at intervals of up to 4 years, with a favourable outcome defined by the absence of symptoms and radiographic evidence of normal periapical structures (ESE 2006). However, the most recent ESE S3‐Level Clinical Practice Guideline (Duncan et al. 2023) no longer establishes a fixed four‐year period; instead, it recommends clinical follow‐up at approximately 6 months and radiographic assessment at 1‐year post‐treatment, with subsequent evaluations as clinically indicated. When healing remains uncertain or incomplete, extended observation periods may be warranted.

Recent literature further emphasises the importance of integrating clinical symptoms with imaging findings and of assessing the risks and benefits of potential retreatment when managing PAP, in order to guide prognosis and treatment planning (Lieblich 2020; Cotti and Schirru 2022; Şanal Çikman et al. 2022; Petean, Gaêta‐Araujo, et al. 2025). Additionally, the interaction between genetic factors and the various stages of apical periodontitis formation and resolution, particularly involving genes associated with inflammation and bone metabolism, has been demonstrated even 1 year after completion of root canal treatment (Petean et al. 2022), supporting the reintervention criteria adopted in the present study. At the same time, it is also worth noting the difficulty of patient adherence to long follow‐up periods, which reinforces the importance of carrying out new studies to replicate the data obtained in larger populations, in different ethnicities (Jakovljevic et al. 2022; Petean et al. 2022; De Castro et al. 2023) and different follow‐up times, as well as new integrated analyses of the effects of host genetics on the development and repair of PAP.

Overview, given their limitations, the data and analysis methodologies used in this exploratory study shed light on new strategies that can be used to establish precision dentistry for the treatment of PAP. This may help to envision the development of new therapies based on the identification of therapeutic targets and the development of materials and techniques that aim to act at a molecular level for the clinical, radiographic, and histological success of RCT.

## 5 Conclusions

Different gene expression values were observed according to the genotypes of the polymorphisms in *SOCS‐1, TNF* and *RANKL* in cases of persistent apical periodontitis. Although the sample size was limited and the results should therefore be interpreted with caution, the findings suggest that genetic polymorphisms may serve as promising biomarkers for post‐treatment apical periodontitis, as they appear to modulate gene expression and protein activity, thereby influencing the host's inflammatory and reparative responses. This functional perspective provides a biologically plausible link between genetic variability and clinical outcomes. Accordingly, the exploratory nature of this investigation provides support for future translational research aimed at advancing personalised endodontic treatment strategies.

## Author Contributions

**Igor Bassi Ferreira Petean:** conceptualisation, methodology, visualisation, investigation, data curation, formal analysis, writing – original draft, writing – review and editing, project administration. **Alice Corrêa Silva‐Sousa:** methodology, project administration. **Rafael Camargo Verardino, Yara Terezinha Corrêa Silva‐Sousa:** methodology. **Fernanda Gonçalves Basso, André Pitondo‐Silva:** investigation, data curation, writing – review and editing. **Francisco Wanderley Garcia de Paula‐Silva:** methodology, visualisation, investigation, data curation. **Erika Calvano Kuchler, Raquel Assed Bezerra Segato, Lea Assed Bezerra da Silva:** methodology. **Jardel Francisco Mazzi‐Chaves, Fabiane Carneiro Lopes‐Olhê:** methodology, project administration. **Manoel Damião Sousa‐Neto:** conceptualisation, methodology, visualisation, investigation, data curation, formal analysis, writing – review and editing, supervision, project administration.

## Conflicts of Interest

The authors declare no conflicts of interest.

## Data Availability

The data that support the findings of this study are available from the corresponding author upon reasonable request.

## References

[iej70070-bib-0002] Alexander, W. S. 2002. “Suppressors of Cytokine Signalling (SOCS) in the Immune System.” Nature Reviews. Immunology 2: 410–416. 10.1038/nri818 12093007

[iej70070-bib-0001] Alexander, W. S. , and D. J. Hilton . 2004. “The Role of Suppressors of Cytokine Signaling (SOCS) Proteins in Regulation of the Immune Response.” Annual Review of Immunology 22: 503–529. 10.1146/annurev.immunol.22.091003.090312 15032587

[iej70070-bib-0003] Aminoshariae, A. , and J. C. Kulild . 2015. “Association of Functional Gene Polymorphism With Apical Periodontitis.” Journal of Endodontics 41: 999–1007. 25952187 10.1016/j.joen.2015.03.007

[iej70070-bib-0004] Bachtiar, B. M. , and B. M. Bachtiar . 2017. “Proinflammatory MG‐63 Cells Response Infection With *Enterococcus faecalis* cps2 Evaluated by the Expression of TLR‐2, IL‐1β, and iNOS mRNA.” BMC Research Notes 10: 401. 10.1186/s13104-017-2740-4. 28800779 PMC5553915

[iej70070-bib-0005] Bek, S. , J. V. Nielsen , A. B. Bojesen , et al. 2016. “Systematic Review: Genetic Biomarkers Associated With Anti‐TNF Treatment Response in Inflammatory Bowel Diseases.” Alimentary Pharmacology & Therapeutics 44, no. 6: 554–567. 27417569 10.1111/apt.13736 PMC5113857

[iej70070-bib-0006] Boonkasemsanti, W. , C. Padungkarn , S. Tewtipsakul , and E. Phattarataratip . 2025. “Periapical Lesions: Assessment of Clinical Diagnostic Accuracy and Prevalence of Nonendodontic Lesions Mimicking Endodontic Pathoses.” International Endodontic Journal. 10.1111/iej.14278 40598469

[iej70070-bib-0007] Bornstein, M. M. , A. C. Bingisser , P. A. Reichart , P. Sendi , D. D. Bosshardt , and T. von Arx . 2015. “Comparison Between Radiographic (2‐Dimensional and 3‐Dimensional) and Histologic Findings of Periapical Lesions Treated With Apical Surgery.” Journal of Endodontics 41: 804–811. 25863407 10.1016/j.joen.2015.01.015

[iej70070-bib-0008] Campos, K. , C. F. Franscisconi , V. Okehie , et al. 2015. “FOXP3 DNA Methylation Levels as a Potential Biomarker in the Development of Periapical Lesions.” Journal of Endodontics 41, no. 2: 212–218. 25459573 10.1016/j.joen.2014.10.003

[iej70070-bib-0009] Cavalla, F. , A. Letra , R. M. Silva , and G. P. Garlet . 2021. “Determinants of Periodontal/Periapical Lesion Stability and Progression.” Journal of Dental Research 100, no. 1: 29–36. 32866421 10.1177/0022034520952341

[iej70070-bib-0010] Chen, S. S. , W. Z. Wu , Y. P. Zhang , and W. J. Huang . 2020. “Gene Polymorphisms of SOCS‐1 and SOCS2 and Acute Lymphoblastic Leukemia.” European Review for Medical and Pharmacological Sciences 24, no. 10: 5564–5572. 32495891 10.26355/eurrev_202005_21342

[iej70070-bib-0011] Cotti, E. , and E. Schirru . 2022. “Present Status and Future Directions: Imaging Techniques for the Detection of Periapical Lesions.” International Endodontic Journal 55, no. 4: 1085–1099. 36059089 10.1111/iej.13828

[iej70070-bib-0012] Couto, A. M. D. , D. P. Meirelles , A. T. Valeriano , et al. 2021. “Chronic Inflammatory Periapical Diseases: A Brazilian Multicenter Study of 10,381 Cases and Literature Review.” Brazilian Oral Research 35: e033. 33729278 10.1590/1807-3107bor-2021.vol35.0033

[iej70070-bib-0013] Crommelin, H. , A. Vorselaars , J. van der Vis , V. Deneer , and C. H. M. van Moorsel . 2020. “Pharmacogenetics of Antitumor Necrosis Factor Therapy in Severe Sarcoidosis.” Current Opinion in Pulmonary Medicine 26, no. 3: 267–276. 32205584 10.1097/MCP.0000000000000681

[iej70070-bib-0014] De Castro, G. A. P. , I. B. F. Petean , F. W. G. de Paula‐Silva , et al. 2023. “Genetic Polymorphism in the Tumour Necrosis Factor Alpha Gene (G‐308A) is Associated With Persistent Apical Periodontitis in Brazilians.” International Endodontic Journal 56, no. 1: 17–26. 36183324 10.1111/iej.13841

[iej70070-bib-0015] De Soyza, A. , A. J. Hall , E. Mahenthiralingam , et al. 2013. “Developing an International *Pseudomonas aeruginosa* Reference Panel.” Microbiology 2, no. 6: 1010–1023. 10.1002/mbo3.141 PMC3892346 24214409

[iej70070-bib-0016] Del Fabbro, M. , S. Corbella , P. Sequeira‐Byron , et al. 2016. “Endodontic Procedures for Retreatment of Periapical Lesions.” Cochrane Database of Systematic Reviews 10, no. 10: CD005511. 27759881 10.1002/14651858.CD005511.pub3 PMC6461161

[iej70070-bib-0017] Dessaune‐Neto, N. , M. T. M. Porpino , H. d. S. Antunes , et al. 2018. “Pro‐Inflammatory and Anti‐Inflammatory Cytokine Expression in Post‐Treatment Apical Periodontitis.” Journal of Applied Oral Science 26: e20170455. 29898177 10.1590/1678-7757-2017-0455 PMC5963913

[iej70070-bib-0018] Dioguardi, M. , C. Stellacci , L. La Femina , et al. 2022. “Comparison of Endodontic Failures Between Nonsurgical Retreatment and Endodontic Surgery: Systematic Review and Meta‐Analysis With Trial Sequential Analysis.” Medicina (Kaunas, Lithuania) 58, no. 7: 894. 35888613 10.3390/medicina58070894 PMC9324533

[iej70070-bib-0019] Duncan, H. F. , L. L. Kirkevang , O. A. Peters , et al. 2023. “Treatment of Pulpal and Apical Disease: The European Society of Endodontology (ESE) S3‐Level Clinical Practice Guideline.” International Endodontic Journal 56, no. 3: 238–295. 37772327 10.1111/iej.13974

[iej70070-bib-0020] Enriquez, F. J. J. , J. P. Vieyra , and F. P. Ocampo . 2015. “Relationship Between Clinical and Histopathologic Findings of 40 Periapical Lesions.” Dentistry 5: 277.

[iej70070-bib-0021] ESE . 2006. “Quality Guidelines for Endodontic Treatment: Consensus Report of the European Society of Endodontology.” International Endodontic Journal 39, no. 12: 921–930. 17180780 10.1111/j.1365-2591.2006.01180.x

[iej70070-bib-0022] Estrela, C. , M. R. Bueno , B. C. Azevedo , J. R. Azevedo , and J. D. Pécora . 2008. “A New Periapical Index Based on Cone Beam Computed Tomography.” Journal of Endodontics 34, no. 11: 1325–1331. 18928840 10.1016/j.joen.2008.08.013

[iej70070-bib-0023] Estrela, C. , R. Holland , C. R. Estrela , A. H. Alencar , M. D. Sousa‐Neto , and J. D. Pécora . 2014. “Characterization of Successful Root Canal Treatment.” Brazilian Dental Journal 25, no. 1: 3–11. 24789284 10.1590/0103-6440201302356

[iej70070-bib-0024] Fouad, A. F. , A. A. Khan , R. M. Silva , and M. K. Kang . 2020. “Genetic and Epigenetic Characterization of Pulpal and Periapical Inflammation.” Frontiers in Physiology 11: 21. 32116745 10.3389/fphys.2020.00021 PMC7010935

[iej70070-bib-0025] Fukushima, A. , H. Kajiya , T. Izumi , C. Shigeyama , K. Okabe , and H. Anan . 2010. “Pro‐Inflammatory Cytokines Induce Suppressor of Cytokine Signaling‐3 in Human Periodontal Ligament Cells.” Journal of Endodontics 36: 1004–1008. 20478455 10.1016/j.joen.2010.02.027

[iej70070-bib-0026] Garlet, G. P. 2010. “Destructive and Protective Roles of Cytokines in Periodontitis: A Re‐Appraisal From Host Defense and Tissue Destruction Viewpoints.” Journal of Dental Research 89, no. 12: 1349–1363. 20739705 10.1177/0022034510376402

[iej70070-bib-0027] Graves, D. T. , T. Oates , and G. P. Garlet . 2011. “Review of Osteoimmunology and the Host Response in Endodontic and Periodontal Lesions.” Journal of Oral Microbiology 8: 5304. 10.3402/jom.v3i0.5304 PMC3087239 21547019

[iej70070-bib-0028] Guimarães, M. R. , F. R. M. Leite , L. C. Spolidorio , K. L. Kirkwood , and C. Rossa . 2013. “Curcumin Abrogates LPS‐Induced Pro‐Inflammatory Cytokines in RAW 264.7 Macrophages. Evidence for Novel Mechanisms Involving SOCS‐1, −3 and p38 MAPK.” Archives of Oral Biology 58: 1309–1317. 24011306 10.1016/j.archoralbio.2013.07.005 PMC4030384

[iej70070-bib-0029] Horiuchi, A. , E. Kokubu , T. Warita , and K. Ishihara . 2020. “Synergistic Biofilm Formation by Parvimonas Micra and *Fusobacterium nucleatum* .” Anaerobe 62: 102100. 31521732 10.1016/j.anaerobe.2019.102100

[iej70070-bib-0030] Jakovljevic, A. , J. Jacimovic , A. C. Georgiou , et al. 2022. “Single Nucleotide Polymorphisms as a Predisposing Factor for the Development of Apical Periodontitis—An Umbrella Review.” International Endodontic Journal 55, no. 7: 700–713. 35476797 10.1111/iej.13756

[iej70070-bib-0031] Jakovljevic, A. , N. Nikolic , J. Carkic , et al. 2020. “Association of Polymorphisms in TNF‐α, IL‐1β, GSTM and GSTT Genes With Apical Periodontitis: Is There a Link With Herpesviral Infection?” International Endodontic Journal 53, no. 7: 895–904. 32216135 10.1111/iej.13298

[iej70070-bib-0032] Jakovljevic, A. , N. Nikolic , J. Jacimovic , et al. 2021. “Tumor Necrosis Factor Alpha −308 G/A Single‐Nucleotide Polymorphism and Apical Periodontitis: An Updated Systematic Review and Meta‐Analysis.” Journal of Endodontics 47, no. 7: 1061–1069. 33775731 10.1016/j.joen.2021.03.007

[iej70070-bib-0033] Kim, S. , and S. Kratchman . 2006. “Modern Endodontic Surgery Concepts and Practice: A Review.” Journal of Endodontics 32, no. 7: 601–623. 10.1016/j.joen.2005.12.010. 16793466

[iej70070-bib-0034] Kristian, S. A. , V. Datta , C. Weidenmaier , et al. 2005. “D‐Alanylation of Teichoic Acids Promotes Group A Streptococcus Antimicrobial Peptide Resistance, Neutrophil Survival, and Epithelial Cell Invasion.” Journal of Bacteriology 187, no. 19: 6719–6725. 16166534 10.1128/JB.187.19.6719-6725.2005 PMC1251589

[iej70070-bib-0035] Küchler, E. C. , J. F. Mazzi‐Chaves , L. S. Antunes , C. Kirschneck , F. Baratto‐Filho , and M. D. Sousa‐Neto . 2018. “Current Trends of Genetics in Apical Periodontitis Research.” Brazilian Oral Research 32, no. 1: e72. 30365613 10.1590/1807-3107bor-2018.vol32.0072

[iej70070-bib-0036] Küchler, E. C. , P. N. Tannure , P. Falagan‐Lotsch , T. S. Lopes , J. M. Granjeiro , and L. M. F. Amorim . 2012. “Buccal Cells DNA Extraction to Obtain High Quality Human Genomic DNA Suitable for Polymorphism Genotyping by PCR‐RFLP and Real‐Time PCR.” Journal of Applied Oral Science 20: 467–471. 23032210 10.1590/S1678-77572012000400013 PMC3881822

[iej70070-bib-0037] Lee, Y. P. , M. J. Hwang , Y. C. Wu , M. J. Lang , Y. H. Wu , and C. P. Chiang . 2021. “Clinicopathological Study of Periapical Scars.” Journal of Dental Sciences 16, no. 4: 1140–1145. 34484581 10.1016/j.jds.2021.05.008 PMC8403787

[iej70070-bib-0038] Lieblich, S. E. 2020. “Current Concepts of Periapical Surgery: 2020 Update.” Oral and Maxillofacial Surgery Clinics of North America 32, no. 4: 571–582. 32912776 10.1016/j.coms.2020.07.007

[iej70070-bib-0039] Little, J. , J. P. Higgins , J. P. Ioannidis , et al. 2009. “STrengthening the REporting of Genetic Association Studies (STREGA)—An Extension of the STROBE Statement.” Genetic Epidemiology 33, no. 7: 581–598. 19278015 10.1002/gepi.20410

[iej70070-bib-0040] Louis, E. , D. Franchimont , A. Piron , et al. 1998. “Tumour Necrosis Factor (TNF) Gene Polymorphism Influences TNF‐Alpha Production in Lipopolysaccharide (LPS)‐Stimulated Whole Blood Cell Culture in Healthy Humans.” Clinical and Experimental Immunology 113, no. 3: 401–406. 9737669 10.1046/j.1365-2249.1998.00662.x PMC1905064

[iej70070-bib-0041] Low, K. M. T. , K. Dula , W. Bürgin , and T. von Arx . 2008. “Comparison of Periapical Radiography and Limited Cone‐Beam Tomography in Posterior Maxillary Teeth Referred for Apical Surgery.” Journal of Endodontics 34: 557–562. 18436034 10.1016/j.joen.2008.02.022

[iej70070-bib-0042] Maloney, W. J. , and M. A. Weinberg . 2008. “Implementation of the American Society of Anesthesiologists Physical Status Classification System in Periodontal Practice.” Journal of Periodontology 79: 1124–1126. 18597592 10.1902/jop.2008.070625

[iej70070-bib-0043] McElvania Tekippe, E. , S. Shuey , D. W. Winkler , M. A. Butler , and C. A. Burnham . 2013. “Optimizing Identification of Clinically Relevant Gram‐Positive Organisms by Use of the Bruker Biotyper Matrix‐Assisted Laser Desorption Ionization‐Time of Flight Mass Spectrometry System.” Journal of Clinical Microbiology 51, no. 5: 1421–1427. 23426925 10.1128/JCM.02680-12 PMC3647943

[iej70070-bib-0044] Menezes, R. , T. P. Garlet , A. Letra , et al. 2008. “Differential Patterns of Receptor Activator of Nuclear Factor Kappa B Ligand/Osteoprotegerin Expression in Human Periapical Granulomas: Possible Association With Progressive or Stable Nature of the Lesions.” Journal of Endodontics 34: 932–938. 18634923 10.1016/j.joen.2008.05.002 PMC2719712

[iej70070-bib-0045] Menezes, R. , T. P. Garlet , A. P. F. Trombone , et al. 2008. “The Potential Role of Suppressors of Cytokine Signaling in the Attenuation of Inflammatory Reaction and Alveolar Bone Loss Associated With Apical Periodontitis.” Journal of Endodontics 34: 1480–1484. 19026878 10.1016/j.joen.2008.09.003 PMC2719713

[iej70070-bib-0046] Monaghan, L. , S. Jadun , and J. Darcey . 2019. “Endodontic Microsurgery. Part One: Diagnosis, Patient Selection and Prognoses.” British Dental Journal 226, no. 12: 940–948. 31253911 10.1038/s41415-019-0415-3

[iej70070-bib-0047] Morita, Y. , T. Naka , Y. Kawazoe , et al. 2000. “Signals Transducers and Activators of Transcription (STAT)‐Induced STAT Inhibitor‐1 (SSI‐1)/Suppressor of Cytokine Signaling‐1 (SOCS‐1) Suppresses Tumor Necrosis Factor Alpha‐Induced Cell Death in Fibroblasts.” Proceedings of the National Academy of Sciences 97: 5405–5410. 10.1073/pnas.090084797 PMC25841 10792035

[iej70070-bib-0080] Murray, P. R ., E. J. Baron , J. H. Jorgensen , M. L. Landry , and M. A. Pfaller . 2007. *Manual of Clinical Microbiology* . 9th ed. American Society for Microbiology.

[iej70070-bib-0048] Nagendrababu, V. , P. E. Murray , R. Ordinola‐Zapata , et al. 2021. “PRILE 2021 Guidelines for Reporting Laboratory Studies in Endodontology: A Consensus‐Based Development.” International Endodontic Journal 54, no. 9: 1482–1490. 33938010 10.1111/iej.13542

[iej70070-bib-0049] Nakagawa, R. , T. Naka , H. Tsutsui , et al. 2002. “SOCS‐1 Participates in Negative Regulation of LPS Responses.” Immunity 17: 677–687. 12433373 10.1016/s1074-7613(02)00449-1

[iej70070-bib-0050] Nikolic, N. , A. Jakovljevic , J. Carkic , et al. 2019. “Notch Signaling Pathway in Apical Periodontitis: Correlation With Bone Resorption Regulators and Proinflammatory Cytokines.” Journal of Endodontics 45, no. 2: 123–128. 30580840 10.1016/j.joen.2018.10.015

[iej70070-bib-0051] Ørstavik, D. , K. Kerekes , and H. M. Eriksen . 1986. “The Periapical Index: A Scoring System for Radiographic Assessment of Apical Periodontitis.” Endodontics & Dental Traumatology 2: 20–24. 3457698 10.1111/j.1600-9657.1986.tb00119.x

[iej70070-bib-0052] Penesis, V. A. , P. I. Fitzgerald , M. I. Fayad , C. S. Wenckus , E. A. BeGole , and B. R. Johnson . 2008. “Outcome of One‐Visit and Two‐Visit Endodontic Treatment of Necrotic Teeth With Apical Periodontitis: A Randomized Controlled Trial With One‐Year Evaluation.” Journal of Endodontics 34, no. 3: 251–257. 18291270 10.1016/j.joen.2007.12.015

[iej70070-bib-0053] Petean, I. B. F. , H. Gaêta‐Araujo , J. F. Mazzi‐Chaves , et al. 2025. “Clinical and Imaging Aspects Associated With Persistent Apical Periodontitis: Subsides for the Treatment Decision‐Making Process.” Clinical Oral Investigations 29, no. 1: 71. 39836228 10.1007/s00784-024-06132-0

[iej70070-bib-0054] Petean, I. B. F. , E. C. Küchler , I. M. V. Soares , et al. 2019. “Genetic Polymorphisms in RANK and RANKL Are Associated With Persistent Apical Periodontitis.” Journal of Endodontics 45, no. 5: 526–531. 30871729 10.1016/j.joen.2018.10.022

[iej70070-bib-0055] Petean, I. B. F. , A. C. Silva‐Sousa , T. J. Cronenbold , et al. 2022. “Genetic, Cellular and Molecular Aspects Involved in Apical Periodontitis.” Brazilian Dental Journal 33, no. 4: 1–11. 10.1590/0103-6440202205113 PMC9645190 36043561

[iej70070-bib-0056] Petean, I. B. F. , A. C. Silva‐Sousa , G. A. Marañón‐Vásquez , et al. 2025. “Interaction Between Polymorphisms in TNF‐⍺ and RANKL Genes Is Associated With the Development of Persistent Apical Periodontitis, in Brazilian Subjects.” Archives of Oral Biology 169: 106106. 39426312 10.1016/j.archoralbio.2024.106106

[iej70070-bib-0057] Rakesh, K. , and D. K. Agrawal . 2005. “Controlling Cytokine Signaling by Constitutive Inhibitors.” Biochemical Pharmacology 70: 649–657. 15936728 10.1016/j.bcp.2005.04.042

[iej70070-bib-0058] Ranade, K. , M. S. Chang , C. T. Ting , et al. 2001. “High‐Throughput Genotyping With Single Nucleotide Polymorphisms.” Genome Research 11, no. 7: 1262–1268. 11435409 10.1101/gr.157801 PMC311112

[iej70070-bib-0059] Ricucci, D. , and J. F. Siqueira Jr. 2010. “Biofilms and Apical Periodontitis: Study of Prevalence and Association With Clinical and Histopathologic Findings.” Journal of Endodontics 36, no. 8: 1277–1288. 20647081 10.1016/j.joen.2010.04.007

[iej70070-bib-0060] Rider, D. , H. Furusho , S. Xu , et al. 2016. “Elevated CD14 (Cluster of Differentiation 14) and Toll‐Like Receptor (TLR) 4 Signaling Deteriorate Periapical Inflammation in TLR2 Deficient Mice.” Anatomical Record 299, no. 9: 1281–1292. 10.1002/ar.23383. PMC4982827 27314637

[iej70070-bib-0061] Sadikot, R. T. , T. S. Blackwell , J. W. Christman , and A. S. Prince . 2005. “Pathogen–Host Interactions in *Pseudomonas aeruginosa* Pneumonia.” American Journal of Respiratory and Critical Care Medicine 171, no. 11: 1209–1223. 15695491 10.1164/rccm.200408-1044SO PMC2718459

[iej70070-bib-0062] Şanal Çikman, A. , T. E. Köse , D. N. Günaçar , E. Çene , and B. Arıcıoğlu . 2022. “Evaluation of Endodontically Treated Teeth and Related Apical Periodontitis Using Periapical and Endodontic Status Scale: Retrospective Cone‐Beam Computed Tomography Study.” Australian Endodontic Journal 48, no. 3: 431–443. 35690589 10.1111/aej.12638

[iej70070-bib-0064] Setzer, F. C. , M. R. Kohli , S. B. Shah , B. Karabucak , and S. Kim . 2012. “Outcome of Endodontic Surgery: A Meta‐Analysis of the Literature—Part 2: Comparison of Endodontic Microsurgical Techniques With and Without the Use of Higher Magnification.” Journal of Endodontics 38: 1–10. 22152611 10.1016/j.joen.2011.09.021

[iej70070-bib-0065] Signor, B. , L. C. Blomberg , P. M. P. Kopper , et al. 2021. “Root Canal Retreatment: A Retrospective Investigation Using Regression and Data Mining Methods for the Prediction of Technical Quality and Periapical Healing.” Journal of Applied Oral Science 29: e20200799. 33886941 10.1590/1678-7757-2020-0799 PMC8075292

[iej70070-bib-0067] Siqueira, J. F. , and I. N. Roças . 2008. “Clinical Implications and Microbiology of Bacterial Persistence After Treatment Procedures.” Journal of Endodontics 34: 1291–1301. 18928835 10.1016/j.joen.2008.07.028

[iej70070-bib-0070] Siqueira, J. F., Jr. , I. N. Roças , D. Ricucci , and M. Hülsmann . 2014. “Causes and Management of Post‐Treatment Apical Periodontitis.” British Dental Journal 216, no. 6: 305–312. 24651336 10.1038/sj.bdj.2014.200

[iej70070-bib-0068] Siqueira, J. F., Jr. , and I. N. Rôças . 2022. “Present Status and Future Directions: Microbiology of Endodontic Infections.” International Endodontic Journal 55, no. 3: 512–530. 34958494 10.1111/iej.13677

[iej70070-bib-0069] Siqueira, J. F., Jr. , I. N. Rôças , J. C. Provenzano , and B. P. Guilherme . 2011. “Polymorphism of the FcγRIIIa Gene and Post‐Treatment Apical Periodontitis.” Journal of Endodontics 37, no. 10: 1345–1348. 21924179 10.1016/j.joen.2011.06.025

[iej70070-bib-0071] Siqueira Junior, J. F. , I. D. N. Rôças , M. F. Marceliano‐Alves , A. R. Pérez , and D. Ricucci . 2018. “Unprepared Root Canal Surface Areas: Causes, Clinical Implications, and Therapeutic Strategies.” Brazilian Oral Research 32, no. 1: e65. 30365606 10.1590/1807-3107bor-2018.vol32.0065

[iej70070-bib-0072] Sousa‐Neto, M. D. , Y. C. Silva‐Sousa , J. F. Mazzi‐Chaves , et al. 2018. “Root Canal Preparation Using Micro‐Computed Tomography Analysis: A Literature Review.” Brazilian Oral Research 32, no. 1: e66. 30365607 10.1590/1807-3107bor-2018.vol32.0066

[iej70070-bib-0073] Starr, R. , T. A. Willson , E. M. Viney , et al. 1997. “A Family of Cytokine‐Inducible Inhibitors of Signalling.” Nature 387: 917–921. 9202125 10.1038/43206

[iej70070-bib-0074] Stashenko, P. , S. M. Yu , and C. Y. Wang . 1992. “Kinetics of Immune Cell and Bone Resorptive Responses to Endodontic Infections.” Journal of Endodontics 18: 422–426. 9796508 10.1016/S0099-2399(06)80841-1

[iej70070-bib-0075] Stueland, H. , D. Ørstavik , and T. Handal . 2023. “Treatment Outcome of Surgical and Non‐Surgical Endodontic Retreatment of Teeth With Apical Periodontitis.” International Endodontic Journal 56, no. 6: 686–696. 36938637 10.1111/iej.13914

[iej70070-bib-0076] Sullivan, M. , G. Gallagher , and V. Noonan . 2016. “The Root of the Problem: Occurrence of Typical and Atypical Periapical Pathoses.” Journal of the American Dental Association 147, no. 8: 646–649. 27046538 10.1016/j.adaj.2016.02.018

[iej70070-bib-0077] Trombone, A. P. F. , F. Cavalla , E. M. V. Silveira , et al. 2016. “MMP1‐1607 Polymorphism Increases the Risk for Periapical Lesion Development Through the Upregulation MMP‐1 Expression in Association With Pro‐Inflammatory Milieu Elements.” Journal of Applied Oral Science 24: 366–375. 27556208 10.1590/1678-775720160112 PMC4990366

[iej70070-bib-0078] Venskutonis, T. , G. Plotino , L. Tocci , G. Gambarini , J. Maminskas , and G. Juodzbalys . 2015. “Periapical and Endodontic Status Scale Based on Periapical Bone Lesions and Endodontic Treatment Quality Evaluation Using Cone‐Beam Computed Tomography.” Journal of Endodontics 41: 190–196. 25498834 10.1016/j.joen.2014.10.017

[iej70070-bib-0079] Wang, S.‐M. , N. Ma , L.‐H. Qiu , X.‐L. Li , D. Yang , and M. Xue . 2017. “Expression of SOCS‐1 and SOCS‐3 in Chronic Periapical Lesions and Its Clinical Significance.” Shanghai Kou Qiang Yi Xue 26: 384–388. 29199331

